# Inter-species cortical registration between macaques and humans using a functional network property under a spherical demons framework

**DOI:** 10.1371/journal.pone.0258992

**Published:** 2021-10-21

**Authors:** Haewon Nam, Chongwon Pae, Jinseok Eo, Maeng-Keun Oh, Hae-Jeong Park

**Affiliations:** 1 Department of Liberal Arts, Hongik University, Sejong, Republic of Korea; 2 Department of Nuclear Medicine, Yonsei University College of Medicine, Seoul, Republic of Korea; 3 Center for Systems and Translational Brain Sciences, Institute of Human Complexity and Systems Science, Yonsei University, Seoul, Republic of Korea; 4 Department of Nuclear Medicine, Department of Psychiatry, Yonsei University College of Medicine, Seoul, Republic of Korea; 5 Graduate School of Medical Science, Brain Korea 21 Project, Yonsei University College of Medicine, Seoul, Republic of Korea; 6 Department of Cognitive Science, Yonsei University, Seoul, Republic of Korea; University of North Carolina at Chapel Hill, UNITED STATES

## Abstract

Systematic evaluation of cortical differences between humans and macaques calls for inter-species registration of the cortex that matches homologous regions across species. For establishing homology across brains, structural landmarks and biological features have been used without paying sufficient attention to functional homology. The present study aimed to determine functional homology between the human and macaque cortices, defined in terms of functional network properties, by proposing an iterative functional network-based registration scheme using surface-based spherical demons. The functional connectivity matrix of resting-state functional magnetic resonance imaging (rs-fMRI) among cortical parcellations was iteratively calculated for humans and macaques. From the functional connectivity matrix, the functional network properties such as principal network components were derived to estimate a deformation field between the human and macaque cortices. The iterative registration procedure updates the parcellation map of macaques, corresponding to the human connectome project’s multimodal parcellation atlas, which was used to derive the macaque’s functional connectivity matrix. To test the plausibility of the functional network-based registration, we compared cortical registration using structural versus functional features in terms of cortical regional areal change. We also evaluated the interhemispheric asymmetry of regional area and its inter-subject variability in humans and macaques as an indirect validation of the proposed method. Higher inter-subject variability and interhemispheric asymmetry were found in functional homology than in structural homology, and the assessed asymmetry and variations were higher in humans than in macaques. The results emphasize the significance of functional network-based cortical registration across individuals within a species and across species.

## Introduction

Inter-species cortical registration between humans and macaques is an essential step in systematically evaluating cross-species commonalities and differences and applying preclinical results to human applications. Compared with the matching of the subcortical structures, matching the cortex across species is challenging because the cortical regions are not explicitly distinguished and because inter-species differences may be more significant in the cortex than in any other brain subsystems. As registration is a procedure that maps homology across brains, the features used to match in the registration algorithm define what the homology implies between brains. If we utilize structural features, such as cortical thickness and curvature, homology is defined in the structural or anatomical perspective. As cortical features (e.g., cortical thickness) are better represented over the cortical surface than by volume [1–3], surface-based registration has widely been used to register the cortical features across humans [4–7]. Based on this structural homology, diverse cortical properties such as myelination [7, 8], metabolic activity [9, 10], and tau and amyloid positron emission tomography scans [11] have been evaluated across subjects or groups. In inter-species registration, although not much researched, only structural properties (either macroscopic or microscopic) have mostly been used in previous studies [12–15].

Despite the prevalence of matching structural homology, recent studies have implicated gaps between structural and functional homologies across humans [16, 17], which is an important issue in brain science [18]. Accordingly, the interest in determining functional homology has been increasing in recent brain science studies. In brain network science, which views the brain as a complex network system, functional homology can be defined in terms of functional connectivity or functional networks. For example, Conroy, Singer [19] reported better inter-subject registration of task data using functional connectivity. Inter-subject registration has also been conducted with respect to the brain’s functional properties, particularly functional connectivity of rs-fMRI [20–23]. Robinson, Jbabdi [24] combined functional connectivity properties with other multimodal features such as myelin map or discrete areal delineation to determine inter-subject homology using human cortices’ registration.

Functional homology may well be emphasized in the inter-species registration between humans and macaques. Most studies on inter-species registration have used macroscopic structural features (thickness or sulcus/gyrus landmarks) to define inter-species homology [12, 15]. However, not many attempts have been made to match functional correspondence between humans and macaques, particularly functional connectivity. We hypothesized that structural homology does not sufficiently reflect functional diversity across species; therefore, functional homology, defined explicitly in terms of functional connectivity at each cortical region, would be advantageous for linking different species.

In this study, we defined inter-species homology in terms of resting-state functional network properties. Instead of using a simple functional connectivity metric, we employed graph-theoretic features such as functional node degrees and principal graph components to determine functional homologies across human and macaque cortices. To derive graph-theoretic features that are compatible across species, a parcellation map shared by both species is needed for network construction, which is not available for public use. Therefore, we proposed a framework for inter-species functional registration by iteratively estimating a registration function between human and macaque cortices and constructing a cortical parcellation map of macaques using the spherical demons registration algorithm. The parcellation map for the macaque cortex was initially constructed based on the previous deformation field of structural features. It was again used to determine the deformation field between the two species based on functional network features during registration.

Upon establishing a functional registration framework, we compared registrations using structural features versus functional features in inter-subject variabilities. We also evaluated interhemispheric asymmetry between the left and right hemispheres, inter-subject variabilities, and group differences between macaques and humans. Interhemispheric asymmetry has been known in humans functionally and structurally [25–29]. As interhemispheric asymmetry has been widely researched in the human brain, we used this property in humans and between humans and macaques to validate the proposed method.

We hypothesized functional homology would be more heterogeneous than structurally defined homology. We also hypothesized that humans would show higher inter-subject variability and high interhemispheric asymmetry than macaques. All these evaluations are expected to emphasize the significance of functional network-based cortical registration across individuals within a species and across species.

## Methods

### Background: Diffeomorphic spherical demons

We adopted the spherical demons registration algorithm proposed by Yeo, Sabuncu [30] to register two spherical representations of a hemisphere. The spherical registration goal in this study is to find the optimal transformation *T* between two spherical representations for brain surfaces *S*_*M*_ and *S*_*F*_ such that *S*_*M*_∘*T* is aligned to *S*_*F*_ with respect to functional network features.

The initial diffeomorphic demons algorithm proposed by Vercauteren [31] has been used to find nonlinear diffeomorphic transformation function (or deformation field) *T* between a moving image *I*_*M*_ and a fixed image *I*_*F*_ so that ‖*I*_*M*_∘*T*−*I*_*F*_‖ is minimized. In this diffeomorphic demons algorithm, the hidden transformation *Υ* is introduced to decouple the minimization problem as below:

Step1: Find *T* to minimize ${\left\|I_{M}\circ Τ-I_{F}\right\|}^{2}+\frac{1}{\beta^{2}}{\left\|Τ-\Upsilon \right\|}^{2}$
Step2: Find *Υ* to minimize $\frac{1}{\beta^{2}}{\left\|Τ-\Upsilon \right\|}^{2}+\frac{1}{\delta^{2}}Reg(\Upsilon )$.

Parameters *β* and *δ* balance between the similarity and the regularization costs. The regularization function *Reg* is chosen to regularize the smoothness of *Υ*. The demons algorithm is computationally efficient since step 1, a nonlinear least-squares problem, can be minimized using Gauss-Newton optimization.

In the spherical demons [30], transformation functions *T*, *Υ*:*S*^2^→*S*^2^ are defined on a sphere *S*^2^, and therefore the distance between two transformation functions ‖*T*−*Υ*‖^2^ is represented as the distance between sets of tangent vectors of the transformations *T* and *Υ*. The regularization function is chosen as $Reg(\Upsilon )≜{\left\|\hat{\Upsilon }\right\|}_{V}$, where $\hat{\Upsilon }$ is a restricted deformation on a Hilbert space *V*⊂*H* of vector fields. Thus, the smaller ${\left\|\hat{\Upsilon }\right\|}_{V}$ means to a smoother vector field, consequently the smoother *Υ*.

### Inter-species cortical registration

Let $S_{M}=\{x_{k}\in S^{2}:\;1\leq k\leq N_{M}\}\;\mathrm{and}\;S_{F}=\{x_{k}\in S^{2}:\;1\leq k\leq N_{F}\}$ be two spherical representations of hemispheres of moving (source) sphere and fixed (target) sphere, respectively. For * = *M*, *F*, curvature $C_{*}:S^{2}\to R^{N_{*}}$, sulcus depth $D_{*}:S^{2}\to R^{N_{*}}$, myelin ${My}_{*}:S^{2}\to R^{N_{*}}$ of the white matter surface (structural network features) and rs-fMRI time series sampled at the gray matter region corresponding to every node on each sphere are given. rs-fMRI time series were used to compose functional network features using dual-regression group principal component analysis (PCA). We employ functional network features in each node of the cortical surface to define functional homology across individuals within the same species or between species on the cortical surface.

#### Functional network features from dual-regression group PCA

**Fig 1:**
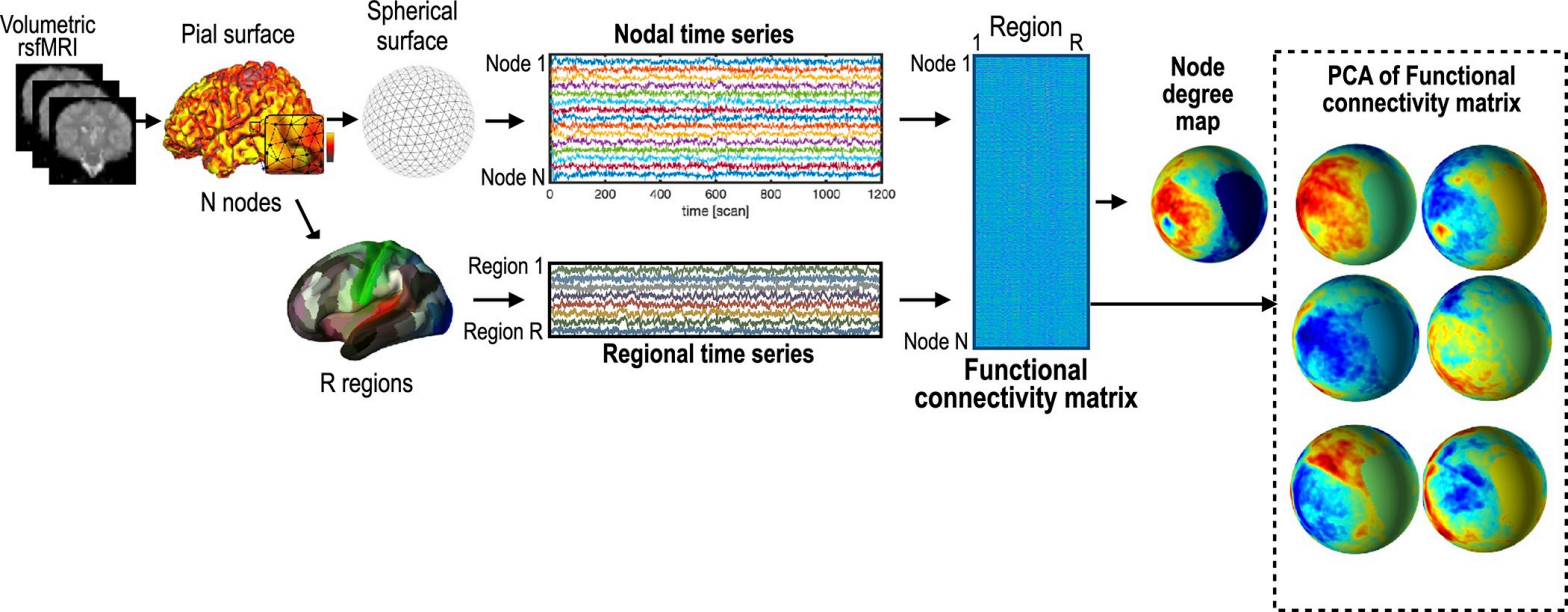
Procedure to extract surface-based resting-state functional connectivity properties. A N nodes x R regions connectivity matrix was constructed by calculating Pearson’s correlation coefficient from all the nodes in the sphere and all the regions in the parcellation map. From the connectivity matrix, functional connectivity features were extracted.

Suppose a cortical parcellation with *R* regions (or regions of interest, ROI) is given on a spherical hemisphere composed of *N* nodes. The element of functional connectivity matrix with size *N*×*R* indicates the correlation between an rs-fMRI time series at a node and a mean rs-fMRI time series of all the nodes at each region in the parcellation map (Fig 1). PCA was applied on a *N*×*R* functional connectivity matrix to reduce the connectivity matrix’s rank.

**Fig 2:**
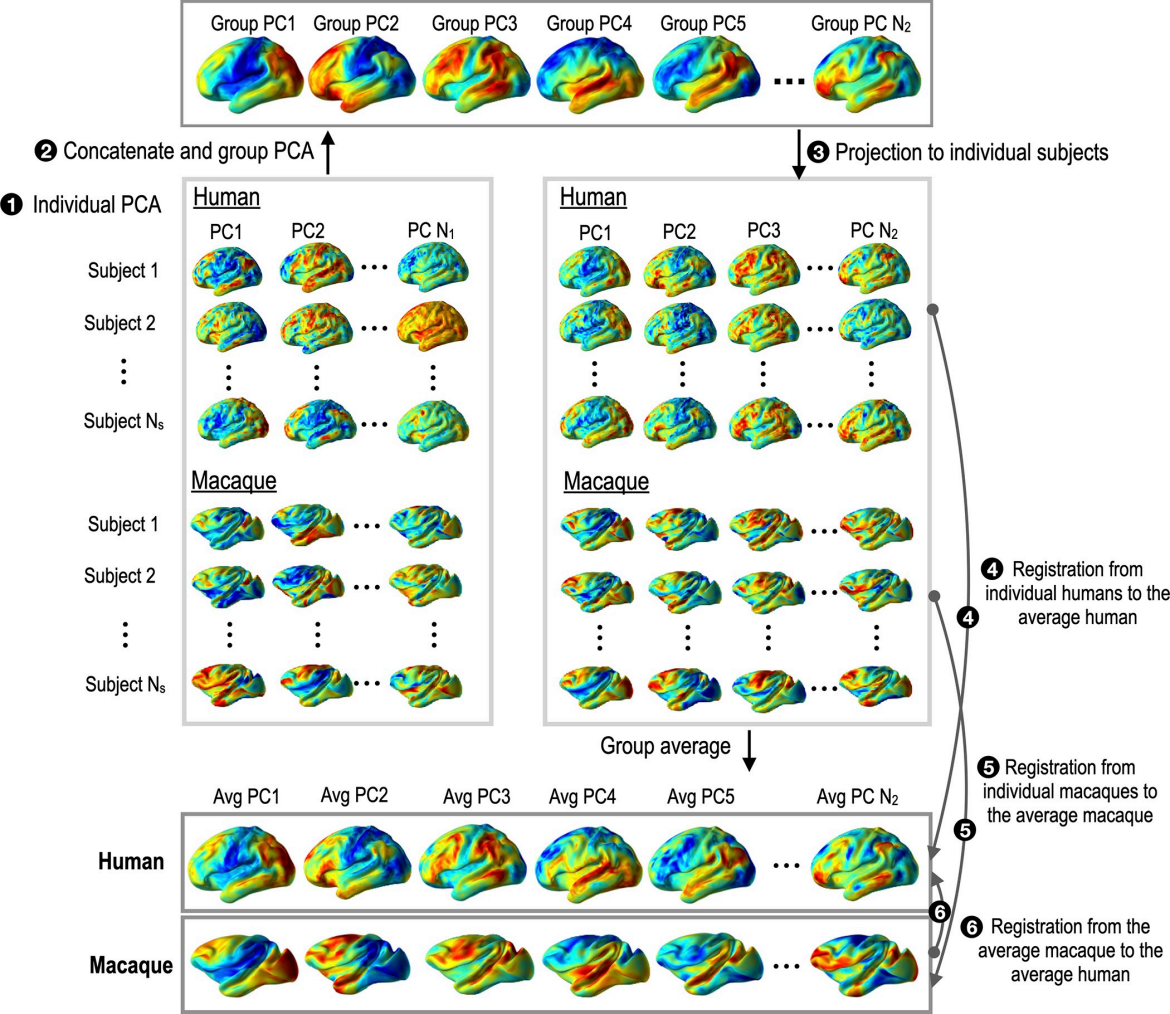
Group PCA procedure of extracting individual principal components (PCs) corresponding to each other and across species as functional connectivity modes. PCA of individual subjects’ PCs was conducted to find group-common PCs in humans and macaques. Those group-common PCs were projected to each individual. These PCs are driven from N x R functional connectivity matrices, not from conventional fMRI time series. From the individual PCs, group-average PCs for humans and macaques were generated. To these group-average PCs, spherical registration of each individual was conducted.

Since principal connectivity (graph) components (PC) derived from PCA (of each individual) differ between species and across individuals, it is not trivial to match the same types of PCs across individuals in the registration. Thus, we used dual-regression group PCA to find individual PCs corresponding to each other. The dual-regression approach has been introduced in the group level independent component analysis [32, 33]. The procedure was explained in Fig 2. Let *N*_*s*_ be the number of subjects in each group. For each subject in each group, PCA was applied to the connectivity matrix and generated *N*_1_ individual PCs (step 1 in Fig 2). We used *N*_1_ = 20 individual PCs, which explains over 90% of the variance. All individual PCs were concatenated to a group PC set (2*N*_*s*_×*N*_1_ *PCs*) for the subsequent group level PCA (step 2 in Fig 2). *N*_2_ = 10 group PCs were generated by the group level PCA of the group PC set of all individual PCs. Among N_2_ = 10 PCs for the group level PCA, we chose six group PCs (*N*_2_ = 6), which has correlation coefficients of 0.5 or higher between human and macaque’s group-average PCs. All group PCs (*N*_2_ = 6) were projected to all individual subjects (step 3 in Fig 2).

**Fig 3:**
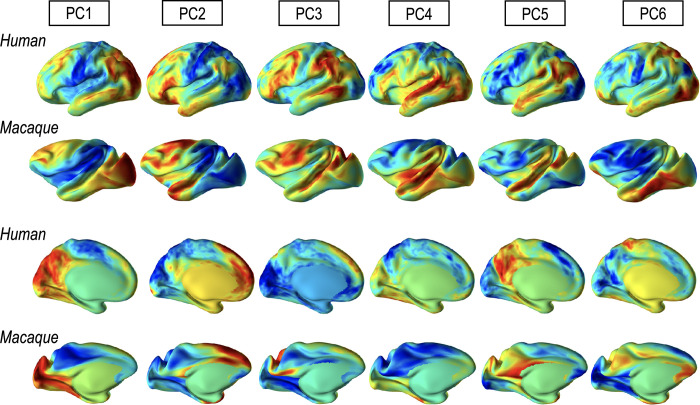
Six group average principal components (modes) of functional connectivity matrix for the human and macaque.

Group-average PCs for macaque and humans were created by averaging projected PCs in each group. All individual PCs in each species were spherically registered to the group-average PCs for the species (steps 4 and 5 in Fig 2). The group-average macaque PCs were registered to those of humans as main functional features (step 6 in Fig 2). Fig 3 shows examples of six group-average PCs for humans and macaques displayed on inflated pial surfaces of human and macaque atlases. The resulting PCs and functional node degree (sum of connectivity with all the other nodes from a node) are used as functional network features in spherical registration. The spherical registration procedures across individuals within each group and registration between species are used in steps 4–6 of Fig 2. The following sections explain details about this spherical registration.

#### Spherical registration with structural features and functional network features; surface vector alignment

For the initial spherical registration, we used curvature, sulcus depth and myelin as structural features to align between target and source spheres (step 1). Humans and macaque have significantly different sulcus patterns, especially in the sensory-motor areas. Thus, sulcus landmarks alone do not provide homology information between humans and macaque, but myelination distribution offers. Therefore, we included myelin distribution in the structural feature. Then, we iteratively registered the source sphere to the target sphere with recursive updates of the macaque’s parcellation map. Suppose that a parcellation map *P*_*F*_ with *R* regions on the target sphere is given. By using a parcellation map *P*_*M*_ corresponding to the parcellation map *P*_*F*_ on the source sphere, we computed functional connectivity matrices with size *N*_*F*_×*R* and *N*_*M*_×*R* for fixed and moving spheres, respectively. Since a common parcellation map for both species is not available, a parcellation map *P*_*M*_ is iteratively estimated by transforming the target parcellation map *P*_*F*_ with the inverse deformation field from the source to the target spheres during the registration process. This will be explained again.

As explained in Fig 2, individual connectivity PCs that match each other across individuals and species were derived by using dual-regression group PCA of functional connectivity matrices. All individual PCs were normalized to be one and weighted with the proportion of corresponding eigenvalues. As a functional connectivity feature, we also included a functional node degree map (the numbers of all brain parcellation regions connected with each node in the whole cortical surface). In the node degree calculation, a threshold to binarize the adjacency matrix was calculated by retaining the top 25% of the connection. The final set of features was composed as below.


$$F=\{C,SD,MY,FD,\lambda_{1}{PC}_{1},\lambda_{2}{PC}_{2},\lambda_{3}{PC}_{3},\lambda_{4}{PC}_{4},\lambda_{5}{PC}_{5},\lambda_{6}{PC}_{6}\}$$

**Fig 4:**
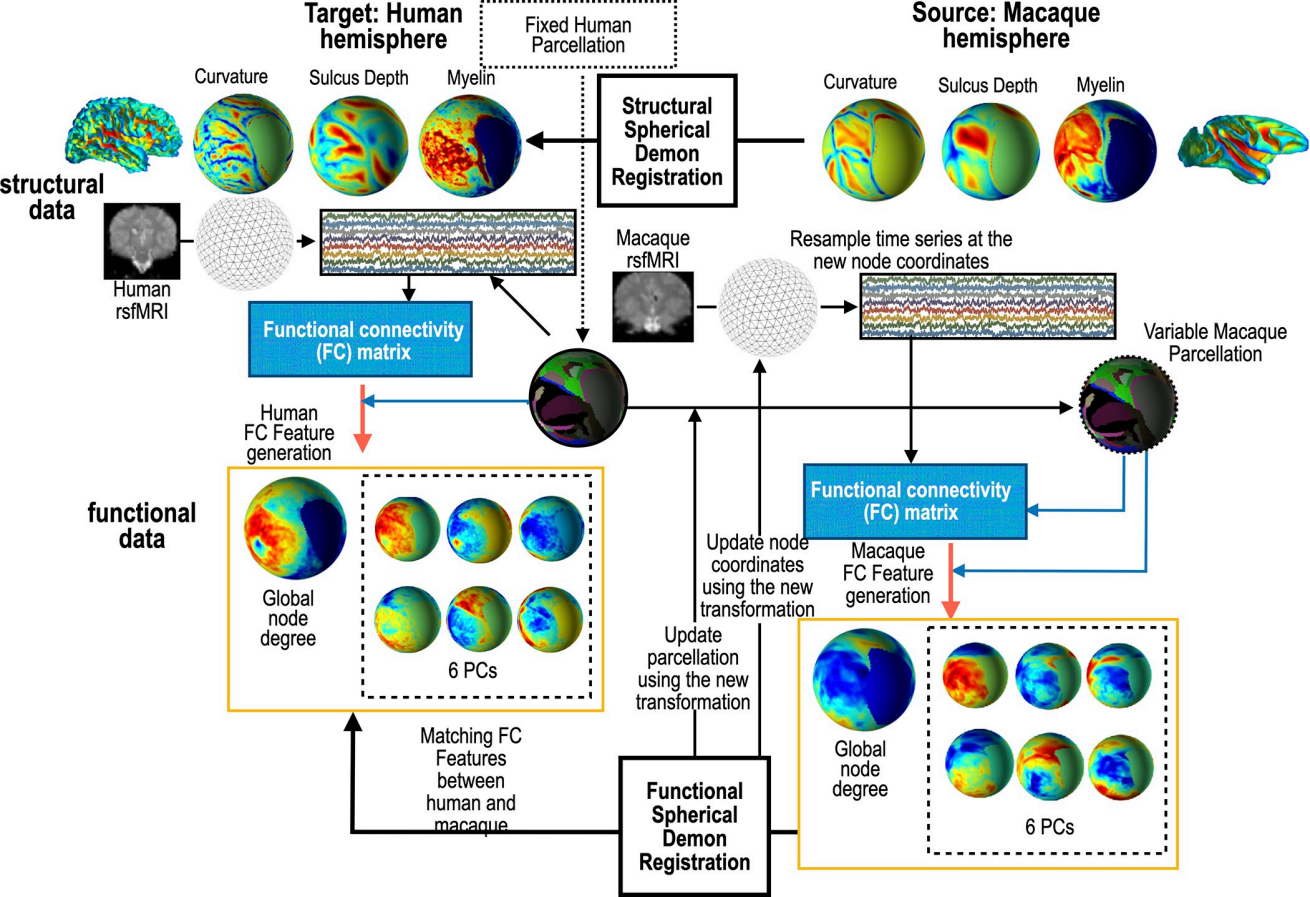
The proposed spherical registration algorithm between macaque and human hemisphere. For each iteration of the proposed algorithm, curvature, sulcus depth, myelination, node degree, and six group principal components based on functional connectivity matrix were generated and used in the demons registration.

C, SD, and MY indicate curvature, sulcal depth, and myelination, FD indicates functional node degree, *λ*_*i*_*PC*_*i*_ indicates i-th weighted PC with coefficient *λ*_*i*_. Throughout this paper, *λ*_*i*_’s was computed based on the ratio of eigenvalues of *PC*_*i*_’s. In this study, 10×*N*_*F*_ feature matrix $F_{F}$ for the fixed sphere and 10×*N*_*M*_ feature matrix $F_{M}$ for the moving sphere were used to define the similarity cost function between the two cortices in the spherical demons algorithm. Details of the proposed registration process is illustrated in Fig 4 and is summarized as follows.

### Step 1—Structural Matching

Find optimal transformation function T using the multi-scale spherical demons registration such that $\left\|F_{M}\circ Τ-F_{F}\right\|$ is minimized. Here, the feature matrices $F_{M}$ and $F_{F}$ are formulated only with structural features: curvature and sulcus depth of the moving and fixed sphere, respectively. The transformation function derived from this structural matching was applied to the parcellation *P*_*F*_ in the *S*_*F*_ to generate an initial parcellation *P*_*M*_ in the moving sphere *S*_*M*_ for functional connectivity matching.

### Step 2—Iterative functional connectivity matching with construction of macaque parcellation map

Set *i* = 1, and *T*_1_ = *T*.

**2.1. *Parcellation map construction*:** Consider that ${F_{M}}_{i}=F_{M}\circ {Τ}_{i}\;\mathrm{and}\;F_{F}$ are aligned in the previous step, apply the parcellation *P*_*F*_ in the *S*_*F*_ to construct a parcellation $P_{M_{i}}$ in the moving sphere ${S_{M}}_{i}$.

**2.2. *Feature extraction*:** Extract fMRI time series at each node and mean fMRI time series at each region in the parcellation map $P_{M_{i}}$ on the moving sphere to compute functional connectivity (cross-correlation between fMRI time series of each node and each region) and to extract functional node degree and weighted PCs at each node in the moving brain. The weight for structural feature is decreasing as the iteration increases, which is denoted *w*_*i*_ in the below equation. We chose $w_{i}=\frac{i}{i+1}$ throughout this paper.


$$F=\{(1-w_{i})\times \{C,SD,MY\},w_{i}\times \{FD,\lambda_{1}{PC}_{1},\lambda_{2}{PC}_{2},\cdots ,\lambda_{6}{PC}_{6}\}\}$$

**2.3. *Registration process*:** Find optimal transformation *T*_*i*+1_ such that $\left\|F_{M_{i}}\circ {Τ}_{i+1}-F_{F}\right\|$ is minimized. The similarity measure in this step includes structural information such as curvature and sulcus depth and functional network information such as node degree, principal components based on parcellation $P_{M_{i}}$. Set *i* = *i*+1. Repeat step 2 until needed.

Finally, a deformation field over the sphere and a parcellation map in the target are derived. The final parcellation map in the moving sphere is derived by applying the deformation field to the fixed sphere’s parcellation map.

In this procedure, we used a cortical parcellation map of the human cortex, subdivided into 180 labels [34]. This cortical parcellation map of the human was morphed into the macaque cortex by utilizing sulcus depth and curvature information in the first step. After calculating cortical parcellation map for each species, resting-state functional connectivity matrix from all the nodes in the cortical surface to all cortical regions in the parcellation map was calculated. We extracted functional network features from the functional connectivity matrix again, which were used spherical registration across species. Utilizing the deformation field from this functional registration, we again morphed the human cortical parcellation map to the macaque cortex space, which was used to evaluate the macaque’s functional connectivity matrix again. We repeated this step several times. We tested whether this procedure updates the cortical parcellation to maximize the functional homology defined by functional network features across species.

### Simulation and experiment with real data

We conducted three analyses. The first analysis was a simulation experiment designed to show the feasibility of the proposed spherical demons algorithm in finding homology between two cortices based on the functional network topological metric. The second analysis was intra-subject interhemispheric registration in humans and macaques. The last analysis was inter-species registration: from macaque to human.

#### Cortical surface representation of structural features and resting-state fMRI network

For the analyses, we used a set of cortical surface representations (spherical surface mesh, curvature, sulcus depth) and rs-fMRI data of 13 subjects from the Human Connectome Project (HCP) database [35]. HCP data includes 7 males/6 females, with average age 30.8 and standard deviation of 4.3. Each cortical surface representation was extracted from T1 weighted MRI by applying freesurfer (https://surfer.nmr.mgh.harvard.edu) [1, 2]. All rs-fMRI data was sampled at 0.72 Hz, with 1200 time points per session during four sessions. The rs-fMRI data were preprocessed according to the HCP minimal preprocessing pipeline [36]. For the analysis of functional network properties (e.g., node degree) at each iteration *i*, we extracted the rs-fMRI time series for vertices corresponding to the HCP’s 180 multi-modal cortical parcellations for each hemisphere [34]. Thirteen cortical surface representations and their correlation matrices were averaged to construct a human brain atlas. These cortical surfaces are aligned to the first cortical surface representation using spherical demons transformation. We used HCP human cortical surfaces of individuals that were pre-registered to the group template in the HCP preprocessing pipeline.

We used rs-fMRI time series from 13 macaques (7 male/6 female, age 5–13 years) acquired at awake resting state from an open resource database [37]. The current study data include ten T1 and rs-fMRI data sets acquired at the vertical Bruker MRI scanner (4.7 Tesla) at Newcastle University Medical School, Institute of Neuroscience for 250 sample scans with TR 2.6 sec (http://fcon_1000.projects.nitrc.org/indi/PRIME/newcastle.html). Of the 13 macaque data sets provided by Newcastle University Medical School, only 9 data sets that have rs-fMRI were included. Four data sets were Siemens Sonata 1.5T and Prisma 3T at Lyon Neuroscience Research Center for 400 sample scans with TR 2.0 sec (http://fcon_1000.projects.nitrc.org/indi/PRIME/crnl.html). The rs-fMRI preprocessing was performed using MNET (an inhouse software for multispecies network analysis toolbox), including realignment, slice-timing, and spatial registration to the template space using DARTEL [38] toolbox, and spatial smoothing of 4x4x4 mm^3^ FWHM.

Since individual cortical surfaces are not available in the macaque, we used structural volumetric registration of individual T1-weighted images to the Yerkes19 T1-weighted macaque image as a macaque template(https://balsa.wustl.edu/reference/show/976nz). We also used the Yerkes19 cortical surfaces as a macaque template surface. All the preprocessed rs-fMRI data were mapped on to the macaque template surface.

#### Simulation for functional connectivity-based spherical registration

**Fig 5:**
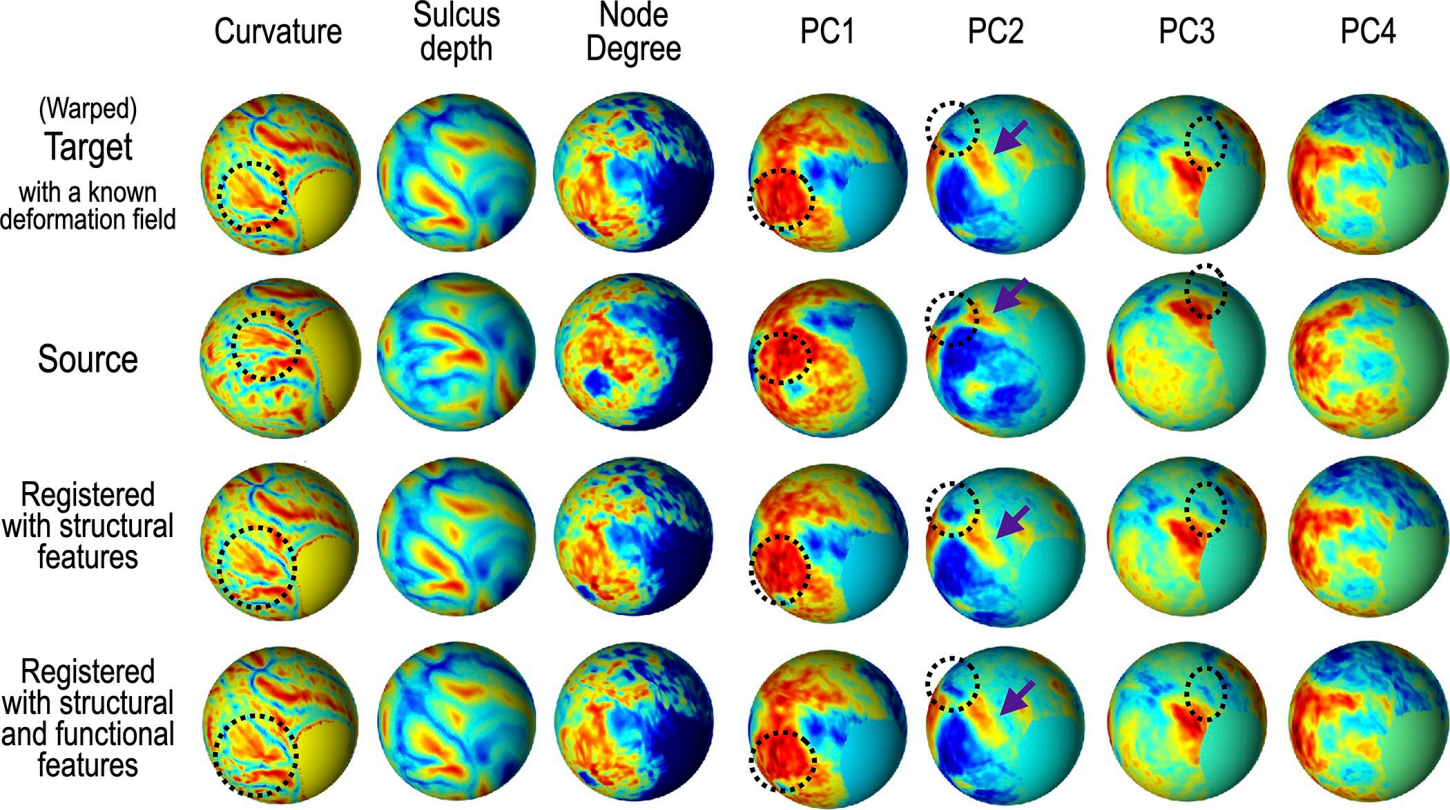
Spherical demons registration results of nonlinear warping simulation. Spheres on the top row show the target features (warped from the source sphere using a ground-truth deformation field). The spheres on the second row show the source features. The last two rows show the registration results only with structural features and with both structural and functional features, respectively. Warping with structural and functional properties makes the source sphere highly aligned with the target sphere.


To test the performance using simulation, we generated a ground-truth deformation field *T*_*G*_, used to warp a sphere *S*. *T*_*G*_ was composed of two consecutive deformations: 1) a combined feature-based *T*_{*C*,*SD*,*MY*}_ by registering two different spheres using structural and functional features and 2) a functional feature-based ${Τ}_{\left\{FD,PC_{i}\right\}}$ which was manually adjusted to induce additional nonlinear deformation at a certain region (Fig 5). The combined feature-based deformation field was applied to warp two different cortical spheres using both structure and functional features of the initial sphere. In the current study, to contrast with structural registration with only the structure features, we denoted registration with the combined features as functional registration. In contrast, the functional feature-based deformation field was further used to warp cortical spheres only using functional features. The final deformation field was a sum of both functional and structure-based deformation fields as below.

$${Τ}_{G}={Τ}_{\left\{C,SD,MY\right\}}\circ {Τ}_{\left\{FD,PC_{i}\right\}}$$

This simulation using a consecutive structural and functional deformation as a ground-truth deformation was conducted to test whether the functional-features are essential to estimate the final deformation field in matching functional homology rather than structural homology. In the first column of Fig 5, dotted circles show where structural and functional-features have deviated from each other.

The resulting warped sphere *S*_*F*_ = *S*∘*T*_*G*_ was considered as a fixed(target) sphere to be inverted to its original sphere using the spherical demons algorithm. We resampled curvature, sulcus depth (structural information), time series, and the parcellation map, according to the deformation field *T*_*G*_. This resampled data plays as a target sphere as a ground truth.

The original *S* was considered as a moving (source) sphere. This simulation was also conducted to validate the proposed algorithm’s convergence after iterations despite misaligned parcellation in the initial guess. Geodesic distance error and the number of invalid parcellated vertices were computed on the sphere for every iteration.

#### Evaluations: Structure versus functional areal changes and their inter-subject variabilities

We compared the structural and functional registration using the areal change index. We define surface area *F*(*S*), the element of which is the sum of the area of all triangles connected to each node in the spherical mesh *S*. The areal change before and after functional registration is defined with areal changing index (ACI), $ACI(S,S_{reg})=\frac{F(S_{reg})-F(S)}{F(S_{reg})+F(S)}$. ACI shows how face area (mesh triangles) in the surface expanded (positive) or shrunk (negative) at each node after registration compared to before registration.

#### Evaluations: Asymmetry and its inter-subject variability

Spherical registration from the right hemisphere to each individual’s left hemisphere in both human and macaque groups was conducted using only structural features and using both structural and functional features separately. After the interhemispheric registration, the interhemispheric brain asymmetry was evaluated by calculating the interhemispheric areal asymmetric index (AAI). The areal change of the right hemisphere before and after functional registration to the left hemisphere is defined with $AAI(S_{left},S_{right})=\frac{F(S_{left})-F(S_{right})}{F(S_{left})+F(S_{right})}$. In this case, *S*_*right*_ is the right hemisphere surface while *S*_*left*_ is the right hemisphere surface registered to the left hemisphere. Positive AAI is the area where the left hemisphere is larger than the right.

#### Inter-species registration and group comparison between macaque and human

For the group study between human and macaque, the cortical surface of individual macaque was registered to the group macaque sphere, followed by transforming it into the HCP cortical surface space using the surface registration function from the macaque to the HCP atlas. Because the human cortical surface has a highly folded morphology compared to that of macaque, maximum of 5 iterations were performed in step 2 to avoid overfitting during surface registration from the macaque cortical surface to the human cortical surface. A two-sample t-test of ACI was conducted to compare group-level differences between macaque and human. To evaluate the asymmetry between groups, we applied a two-sample t-test of absolute AAI, disregarding the hemispheric dominance.

## Results

### Simulation results for functional connectivity-based spherical registration

**Fig 6:**
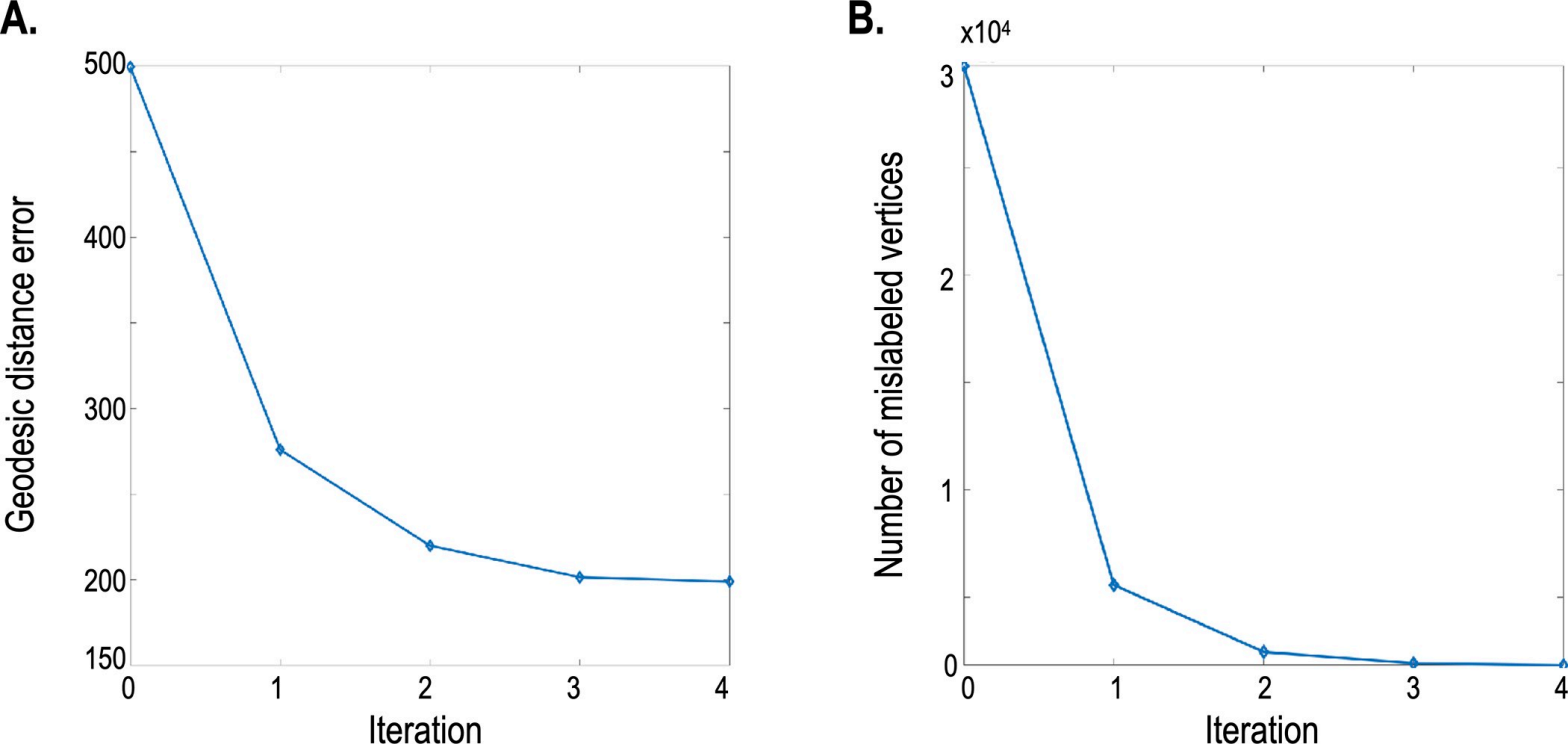
(a) Registration errors defined by the nodes’ geodesic distance are displayed at different iterations. (b) Registration errors defined by the number of wrong-labeled nodes are displayed. The first iteration error was calculated after demons registration only with structural data. In contrast, the registration at the second to the fourth iterations was conducted with both structural and functional properties, weighting functional properties along with the increased iteration.

Fig 5 shows the results of nonlinear warping simulation with an artificially generated deformation field. From top to bottom, each row shows the fixed (target) spheres (which were warped with a ground-truth deformation field), moving (source) spheres, spheres registered with structural features, and spheres registered with both structural and functional network features. From left to right, each row indicates the curvature, sulcus depth, node degree, and first four principal components of the ROI-based connectivity matrix. As the target sphere was nonlinearly registered, the target sphere’s initial parcellation map was highly misaligned. Fig 5 shows dotted circles where the additional warping using a functional deformation field was applied to the initial deformation field using structural features. Spherical demons registration using only structural features aligned two spheres roughly, particularly in the region around the dotted circle. Meanwhile, the registration with functional features enabled a more sophisticated alignment. For quantitative evaluation, error plots were used and are shown in Fig 6. Plot (a) in Fig 6 shows the sum of geodesic differences between the nodes of the target sphere and those of the moving sphere, and plot (b) displays the number of nodes for which parcellation is incorrect. Iteration 0 corresponds to the second row, iteration 1 to the third row, and iteration 4 to the last row of Fig 5. The error plots in Fig 6 show that this algorithm converges well within a few additional iterations after structural registration, although misaligned parcellation was used in the first iteration.

### Functional homology versus structural homology and its inter-subject variability

**Fig 7:**
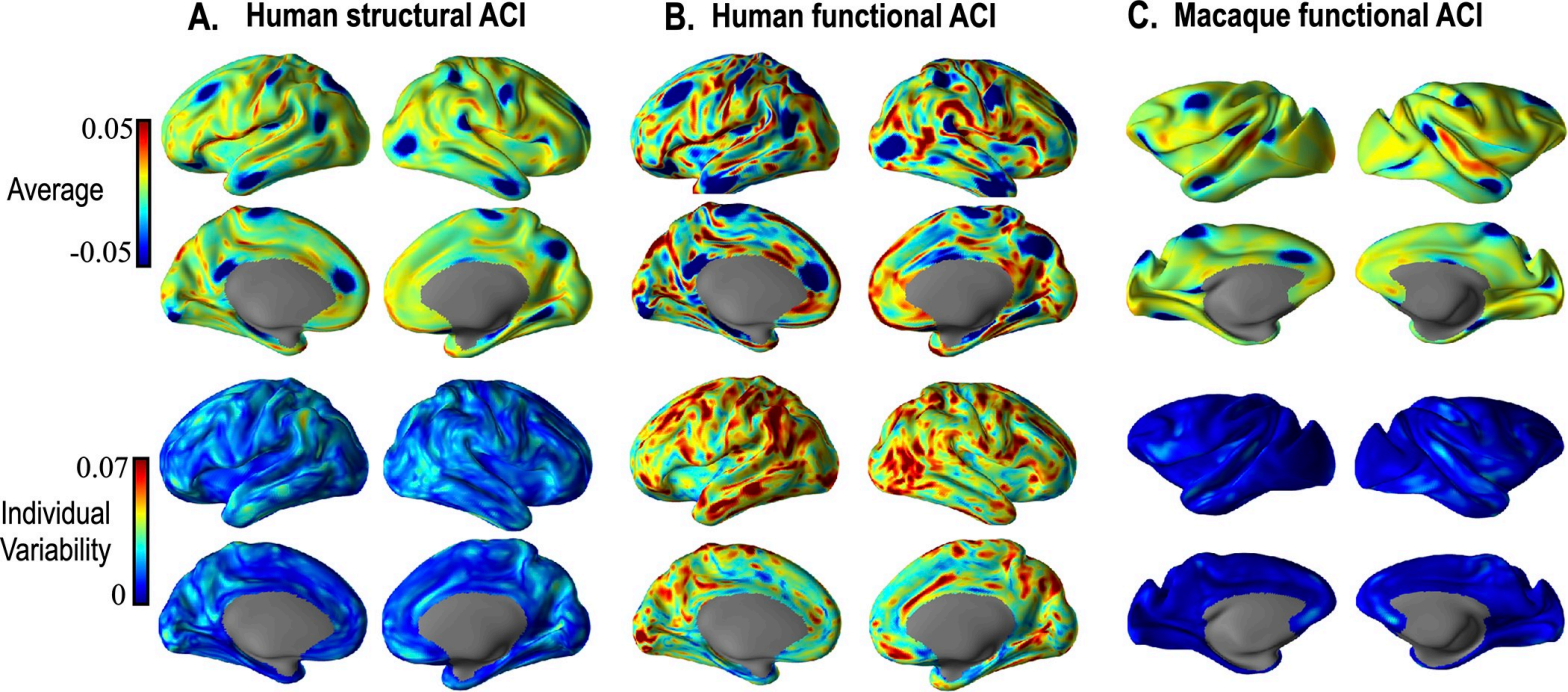
Cortical surface area changes in macaques and humans after structural registration and functional (combined structural and functional features) registration. The left/right hemisphere of human and macaque subjects were registered to the left/right hemisphere of the human template and the macaque template, respectively. The first two top rows are the average of ACI over 13 individuals. The bottom two rows are the standard deviation of ACI. The blue color in the average figure indicates where the area after registration to the group template was less than that of the group template.

Fig 7 shows a comparison of ACI of structural and functional homology in each hemisphere of the human and macaque. Figs 7A, 7B, and 7D show ACI results of each species when registered to its template. Comparing A, B, and C, human ACI by functional registration shows the most considerable individual variabilities. The interindividual variation of structural areal changes is lower than that of functional registration. This is partly because the structural surfaces of the human HCP database were spatially normalized across individuals. The macaque shows more downward inter-subject variations compared to humans.

### Interhemispheric asymmetry and its inter-subject variability

**Fig 8:**
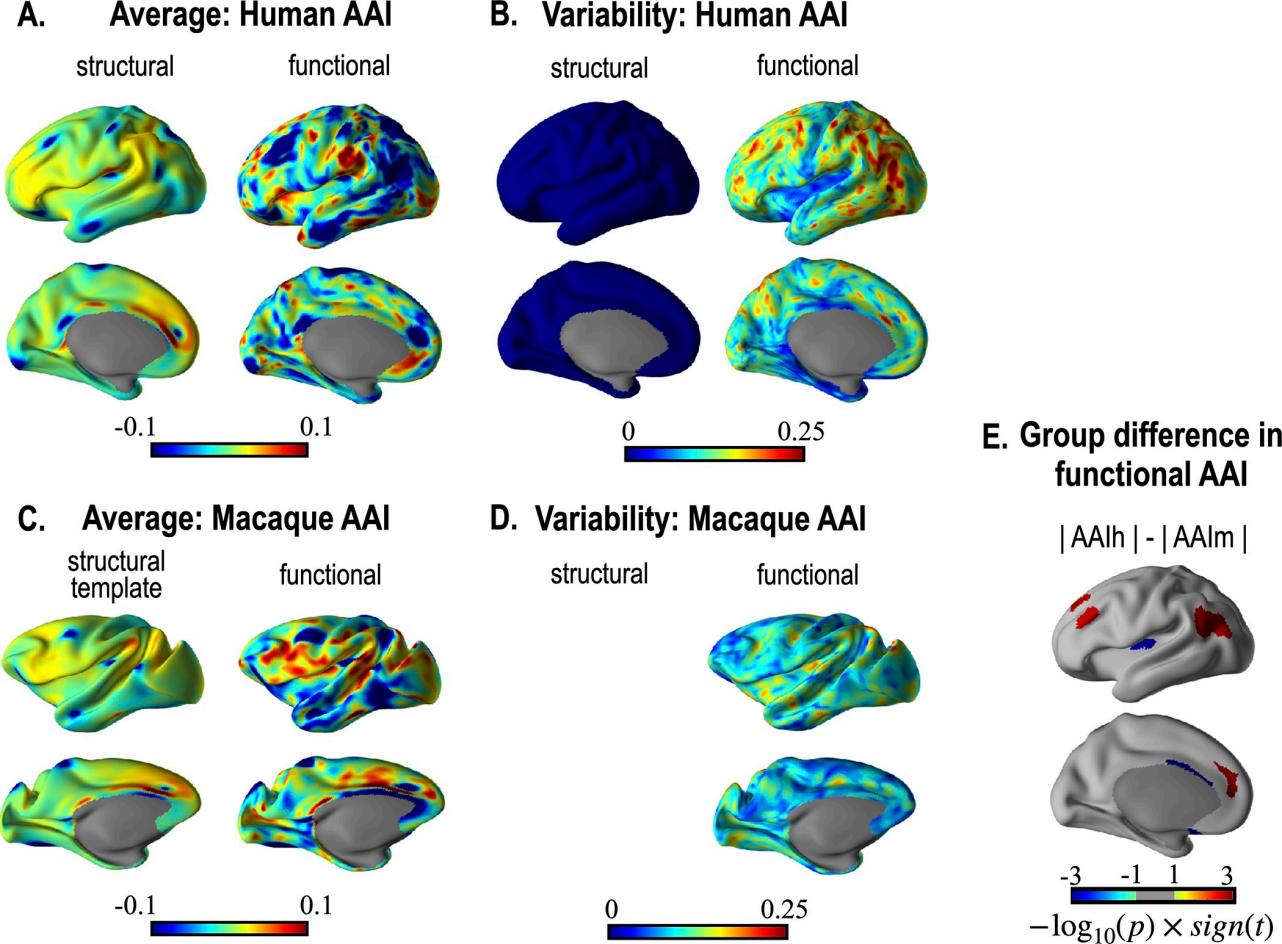
Group interhemispheric asymmetry results: Humans and macaques (13 each). A and B show mean and standard deviation of AAI evaluated after registration of the right hemisphere to the left hemisphere in humans using structural features (left column) and using combined (structural and functional) features (right column) were computed on each parcellation. C and D show the results of the macaque’s interhemispheric registration using structural and functional features. E displays two-sample t-test result of the absolute value of human AAI and macaque AAI with intensity = −log_10_p*sign(*t*).

Fig 8 shows the AAI after registrations of the right to the left hemisphere of the 13 humans and 13 macaques. Fig 8A shows the AAI’s mean over 13 individuals computed on each surface node after structural registration (left column) and functional registration (right column). The area with the positive value (hot colors) was where the left hemisphere was larger than the right hemisphere. Functional registration in humans showed a higher interhemispheric asymmetry than did structural registration. Fig 8B shows standard deviations of the AAI after structural and functional registrations. As shown in Fig 8B, humans’ functional registration showed higher individual variability than structural registration. The low structural variability in the human AAI may be attributable to the fact that HCP cortical surfaces were registered to the template before the current evaluation. Despite the pre-registration across individuals based on structural features, functional homology deviates from the structural homology and shows high inter-individual variations. As we had no individual cortical surfaces for the macaque group, we did not evaluate the inter-subject variability for this group’s structural registration. As presented in the right column of Fig 8B and 8D, humans showed a more heterogeneous interhemispheric asymmetry than macaques. Fig 8E represents two-sample t-tests for the absolute AAI between humans and macaques after functional registration. In Fig 8E, the positive-valued regions (hot colors) indicate the area where humans had a higher AAI than macaques, regardless of hemispheric dominance. Tables 1 and 2 summarize statistical analysis results. Table 1 is the result of significant AAI for each species. Table 2 summarizes group comparison results of AAI between humans and macaques.

**Table 1:** Interhemispheric areal asymmetry.

Macaque					Human				
	mean	std	t	p		mean	std	t	p
MST	-0.116	0.086	-4.878	0.000	V1	-0.014	0.014	-3.557	0.004
V8	-0.057	0.059	-3.467	0.005	V2	-0.035	0.031	-4.043	0.002
POS2	0.038	0.042	3.305	0.006	L_4	-0.040	0.023	-6.128	0.000
PIT	-0.076	0.083	-3.272	0.007	3b	-0.019	0.020	-3.434	0.005
MT	-0.074	0.072	-3.735	0.003	POS1	-0.038	0.039	-3.481	0.005
PSL	0.040	0.047	3.080	0.010	d23ab	-0.156	0.055	-10.303	0.000
v23ab	-0.081	0.068	-4.299	0.001	31pv	-0.084	0.069	-4.415	0.001
d23ab	-0.107	0.035	-11.146	0.000	5L	-0.044	0.043	-3.665	0.003
31pv	-0.071	0.077	-3.302	0.006	LIPv	-0.120	0.132	-3.281	0.007
MIP	-0.106	0.117	-3.271	0.007	VIP	-0.136	0.142	-3.454	0.005
1	-0.080	0.038	-7.555	0.000	MIP	-0.161	0.114	-5.061	0.000
2	-0.049	0.027	-6.539	0.000	1.000	-0.041	0.042	-3.531	0.004
6d	-0.037	0.043	-3.095	0.009	6d	-0.074	0.049	-5.509	0.000
6mp	-0.075	0.039	-6.925	0.000	6mp	-0.125	0.064	-6.997	0.000
33pr	-0.137	0.061	-8.133	0.000	d32	-0.148	0.089	-5.997	0.000
a24	-0.123	0.040	-11.133	0.000	47m	-0.193	0.070	-9.993	0.000
d32	-0.064	0.062	-3.675	0.003	8C	-0.156	0.061	-9.238	0.000
8BM	0.040	0.043	3.366	0.006	47l	-0.101	0.073	-4.981	0.000
47m	-0.227	0.077	-10.597	0.000	p9-46v	-0.102	0.078	-4.686	0.001
8C	-0.111	0.087	-4.591	0.001	OP2-3	-0.129	0.050	-9.343	0.000
47l	-0.070	0.076	-3.326	0.006	52.000	-0.044	0.041	-3.866	0.002
a47r	-0.055	0.024	-8.220	0.000	PoI2	-0.041	0.034	-4.304	0.001
47s	-0.070	0.081	-3.120	0.009	Pir	-0.020	0.021	-3.437	0.005
OP2-3	-0.116	0.117	-3.590	0.004	AAIC	-0.025	0.018	-4.903	0.000
FOP3	0.099	0.099	3.583	0.004	EC	-0.035	0.030	-4.255	0.001
STSda	-0.059	0.056	-3.821	0.002	H	-0.026	0.028	-3.303	0.006
TE1a	-0.118	0.076	-5.650	0.000	PeEc	-0.050	0.035	-5.042	0.000
TE2a	-0.064	0.049	-4.710	0.001	PHA3	-0.063	0.037	-6.226	0.000
IP0	0.032	0.035	3.272	0.007	TE1a	-0.167	0.046	-13.154	0.000
PF	-0.045	0.036	-4.479	0.001	TF	-0.040	0.043	-3.370	0.006
FST	-0.102	0.082	-4.505	0.001	PHT	-0.062	0.067	-3.377	0.006
s32	-0.062	0.068	-3.329	0.006	TPOJ2	-0.087	0.085	-3.670	0.003
pOFC	-0.078	0.051	-5.544	0.000	PGi	-0.137	0.096	-5.146	0.000
p24	-0.057	0.059	-3.470	0.005	PHA2	-0.027	0.032	-3.080	0.010
					PoI1	-0.038	0.032	-4.289	0.001
					LBelt	0.038	0.033	4.200	0.001
					TE1m	-0.056	0.057	-3.537	0.004
					PI	-0.036	0.025	-5.215	0.000

AAI: areal asymmetric index, t: t-value, p: p-value, std: standard deviation. All label names follow Glasser, Coalson [34].

**Table 2:** Group differences in the interhemispheric areal asymmetry between humans and macaques.

Comparison of AAI (H,M)						
	mean(human)	std(human)	mean(monkey)	std(monkey)	t	p
POS2	-0.035	0.067	0.038	0.042	-3.336	0.003
33pr	-0.001	0.049	-0.137	0.061	6.244	0.000
a24	0.048	0.070	-0.123	0.040	7.657	0.000
p9-46v	-0.102	0.078	0.012	0.066	-3.999	0.001
47s	0.018	0.043	-0.070	0.081	3.445	0.002
PGi	-0.137	0.096	-0.028	0.065	-3.379	0.003
25	0.022	0.033	-0.035	0.044	3.723	0.001
s32	0.035	0.065	-0.062	0.068	3.752	0.001
pOFC	0.001	0.026	-0.078	0.051	5.011	0.000
a32pr	-0.036	0.054	0.037	0.054	-3.448	0.002
Comparison of absolute AAI (|H|,|M|)						
	mean(human)	std(human)	mean(monkey)	std(monkey)	t	p
33pr	-0.001	0.049	-0.137	0.061	-5.272	0.000
d32	-0.148	0.089	-0.064	0.062	3.983	0.001
8Ad	-0.008	0.082	0.021	0.039	3.445	0.002
p9-46v	-0.102	0.078	0.012	0.066	3.411	0.002
FOP2	-0.002	0.053	0.067	0.111	-3.404	0.002
TPOJ3	-0.106	0.183	-0.025	0.080	3.537	0.002
PGi	-0.137	0.096	-0.028	0.065	3.907	0.001
pOFC	0.001	0.026	-0.078	0.051	-5.303	0.000
Ig	-0.002	0.029	0.051	0.113	-3.598	0.001

AAI: areal asymmetric index, t: t-value for two-sample t-tests, p: p-value, std: standard deviation. All label names follow Glasser, Coalson [34].

**Fig 9:**
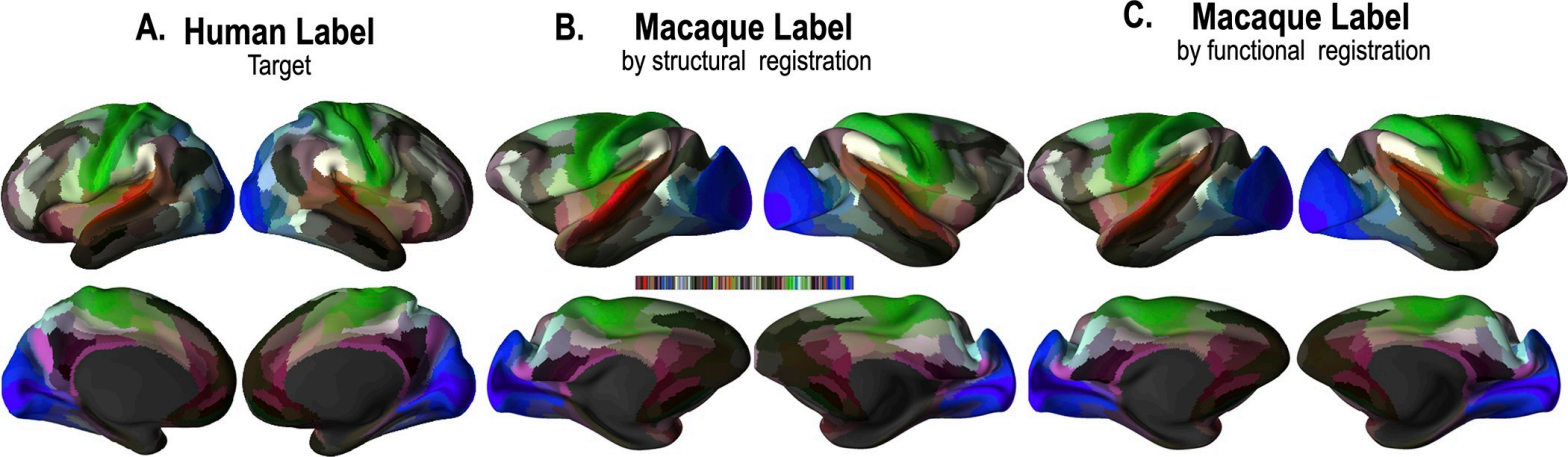
Parcellation maps of the human atlas and macaque atlas. (a) Parcellation map with 180 ROI labels of the human atlas. (b) Parcellation maps on the macaque atlas after registration using only structural features and (c) structural and functional network features.

Fig 9 shows macaque parcellation results after structural and functional registration of the macaque template to the human template. Fig 9A presents the parcellation map of the human HCP template. Fig 9B and 9C show parcellation maps of the macaque template after the registration of structural and functional features, respectively.

## Discussion

Registration is a procedure to determine homology across brains, the meaning of which is reflected in the selection of features for matching. For establishing homology across brains using inter-species registration, structural landmarks such as cortical curvature, sulcus depth, and areal boundaries based on structural MRI have been mainly used [12, 15]. To determine homology across species, structural landmarks alone [13] or in combination with biological properties, such as myelination [39], have been used.

Recent studies have implicated the importance of functional homology across species. Although not many studies are available for inter-species registration, inter-subject registration studies within a group involve the need for functional registration using functional connectivity properties [19–23, 40–42]. In functional registration, the local functional connectivity pattern [21] and within-subject functional connectivity [19, 20] were used. Jiang, Du [21] showed that functional registration improves the resting-state default mode network’s overlap. Nenning, Liu [23] showed that functional registration with resting-state networks improves the statistical power of the group analysis of task fMRI findings. Robinson, Jbabdi [24] used independent component maps of rs-fMRI combined with other multimodal features such as curvature, myelin map, or discrete areal delineation to determine inter-subject homology in cortical registration.

This study proposed an inter-species registration scheme to determine functional homology across humans and macaques using properties of resting-state functional brain networks. We derived the functional connectivity matrix from all nodes in the cortical surface and all regions in the parcellation map to match functional network properties across species. Connectivity between nodes and parcellated regions may be more robust to noise in BOLD signals than connectivity among all nodes. Although we used only a functional node degree metric to describe the functional network property, it can easily be extended to other geometric metrics such as clustering coefficient or local efficiency of a network within each parcellation. It is also possible to use the entire connectivity matrix to determine functional homology across species without deriving abstract metrics. However, to capture principal information in the functional network and minimize noise effects, we applied group PCA to the connectivity matrices. This approach differs from the independent (or principal) component analyses of rs-fMRI time series for generating independent/principal component maps used in the functional registration conducted by Robinson, Jbabdi [24]. The current PCA approach to connectivity matrices is similar to a graph-independent component analysis, which dissolves graphs into multiple independent subgraphs by applying independent component analyses directly to the connectivity matrices [43]. Using PCA to the connectivity matrices, we were able to reduce the complexity in high dimensional feature space during optimization. PCA generates heterogeneous PCs across individuals. To resolve PCs’ correspondence across individuals and species, we used dual-regression PCA, i.e., individual-level PCA of the connectivity matrix, followed by the group-level PCA of the PC components derived from the individual PCA, similarly done in the group-level independent component analysis [32, 33]. By projecting the group PC into the individual level, we could achieve correspondence in graph property patterns across individuals and across species.

For matching functional network properties using the functional connectivity matrix between humans and macaques, a cortical parcellation map in macaques is required, which corresponds to humans. In other words, a common set of network nodes that corresponds one-to-one across species should be determined before matching functional topology. However, to the best of our knowledge, no common representation for network nodes across humans and macaques has been made available to date. Thus, we introduced a method to define a set of network nodes (i.e., a cortical parcellation map) in an iterative manner during functional registration. The scheme alternates between deriving network features based on previous cortical parcellations and redefining cortical parcellations using the deformation field estimated using the network features. Finally, we could derive a cortical parcellation map for the macaque cortex corresponding to that of the human cortex. We confirmed the iterative process utilizing the recursive estimation of functional connectivity metrics and cortical parcellation converges in the simulation (Fig 5), where structural features were insufficient to determine the homologous region. The macaques’ parcellation map will be available for public use in terms of surface mesh and volumetric format.

To test the plausibility for connectivity-based functional registration, we compared the inter-subject variability in the functional homology compared to the structural homology in terms of area changes after registration to the template with functional features compared to structural features. This is based on the common speculation that function homology will show higher inter-individual variations than structural homology. The structural spherical registration served as a baseline for functional registration since the structural spherical registration was conducted on the structurally realigned cortical surfaces in the HCP database. The high inter-subject variability exists in the functional registration, even after structural registration (Fig 7). According to the conventional speculation, the inter-subject variability of functional homology was high only in the human, not in the macaque.

We also evaluated interhemispheric asymmetry and its inter-subject variability in functional homology in terms of regional extent (area) (Fig 8). We found higher asymmetry in functional homology than in structural homology. The inter-subject variability in the interhemispheric cortical asymmetry after functional registration was also higher than that after structural registration. We note that the interhemispheric asymmetry of the regional surface area after registration using structural features in this study may not fully represent the human’s structural asymmetry since we used cortical surfaces of the HCP data spatially normalized to the template. Nevertheless, we confirmed that a higher interhemispheric functional asymmetry remains even after adjusting structural factors using spatial registration to the template space.

The macaque also shows interhemispheric asymmetry in the regional cortical area (Fig 8C). Gannon, Kheck [44] showed that macaques have a higher asymmetry in the cytoarchitectonic region, not in the gross anatomy. Considering that functional connectivity is closer to neuronal distribution (cytoarchitectonic) than gyrus patterns (gross anatomy), the study findings by Gannon, Kheck [44] indirectly support the current result of the higher asymmetry of the functional area than of the structural area.

The inter-subject variability in functional asymmetry was higher in humans than in macaques. This is expected that a higher-order brain system has higher inter-subject variability than a lower-level system in the cognitive hierarchy, exampled in Jang, Knight [45]. Similarly, the human brain has a more complex architecture than that of the macaques, which may explain the higher inter-subject variability in functional interhemispheric asymmetry among human brains than macaques.

Asymmetry in the brain, particularly in the gray and white matter, has been researched [25–29]. Interhemispheric asymmetry in morphology or connectivity may be associated with functional lateralization, such as language or other cognitive domains [46–49]. Xia, Wang [50] studied brain structural asymmetries in developing macaque monkeys from birth to 20 months of age and the leftward increased area at the posterior insula and posterior superior temporal gyrus and ventral occipital cortex, which slightly differs from the current structural asymmetry of the adult macaque in the left-right direction. The current structural asymmetry of the adult macaque was evaluated by the Yerkes19 macaque template (https://balsa.wustl.edu/reference/show/976nz). Although the present study differs from Xia, Wang [50] in the direction, we found a left-lateralized area increase in the lateral, inferior and medial frontal lobes and angular gyrus, similarly to the human (Fig 8A), which are known to be involved in the higher-level information processing. Considering the left-dominancy of the language centers in the human, the left-dominant area in this language region (Broca’s and Wernicke’s areas) may support the current result’s validity.

The asymmetry was highly variable across humans, particularly in the angular gyrus, superior and lateral frontal lobes, compared to the sensory-motor areas (Fig 8B). Meanwhile, the inter-subject variability was relatively low in macaques compared to the human. Van Essen, Donahue [51] reported that humans have the most hemispheric variability and interhemispheric asymmetry among mice, marmosets, macaques, and humans based on structural registration. In their analysis, nearly one-third of all areas (57/180) in the human showed interhemispheric asymmetry (AAI>0.2) by multimodal parcellation. In our analysis of functional connectivity-based registration, 39 regions out of 180 cortical areas had significantly high interhemispheric asymmetry in the human, evaluated after structural registration.

Functional homology defined in the current scheme using rs-fMRI remains validated further using homology defined with task performance. Resting-state functional connectivity may not necessarily correspond to task performance connectivity [52], although there are high overlaps [53–60]. Nevertheless, functional brain connectivity and networks have widely been used to characterize individual characteristics such as maturity [61], character [62], fingerprinting [63], and task performance [64] and to detect individual differences about connectivity [65], cognitive function [66], cognition [67], and clinical symptoms [68]. In line with this research trend, the present study utilized resting-state functional connectivity in determining homology across humans and macaques. We expect the proposed framework of matching functional network properties across species to be highly useful in evaluating cross-species convergence and divergence in the brain functions, which may expand our understanding of humans’ uniqueness.
